# Comparison of 4 kinds of traditional Chinese medicine injections to assist in improving clinical indicators of patients with idiopathic pulmonary fibrosis: A systematic review and network meta-analysis

**DOI:** 10.1097/MD.0000000000031877

**Published:** 2022-11-25

**Authors:** Xiaozheng Wu, Wen Li, Zhong Qin, Zhenliang Luo, Lei Xue, Yunzhi Chen

**Affiliations:** a Department of Preclinical medicine, Guizhou University of Traditional Chinese Medicine, Guiyang, China.

**Keywords:** clinical indicators, idiopathic pulmonary fibrosis, injection, network meta-analysis, safety, traditional Chinese medicine

## Abstract

At present, apart from lung transplantation, no drugs can effectively treat idiopathic pulmonary fibrosis (IPF). Therefore, it is imperative to explore new drugs to control or treat it. Traditional Chinese medicine (TCM) injections have been widely used in the field of IPF, but there is no comparison of their efficacy in the assisted improvement of IPF. Therefore, the purpose of this study is to network meta-analyze the efficacy and safety of 4 kinds of commonly used TCM injections assisted by conventional treatment to improve the disease.

**Methods:**

Used a computer to find the Randomized Controlled Trials (RCTs) from the 8 major databases (PubMed, EMbase, CENTRAL, MEDLINE, CBM, China National Knowledge Infrastructure, WanFang Database and VIP Chinese Science). Cochrane’s risk assessment tool was used to evaluate the quality of the literature. The Grading of Recommendations Assessment, Development and Evaluation approach served to assess the certainty in the evidence of direct and indirect estimates. Revman5.3 (Review Manager (RevMan) Version 5.3. Copenhagen: The Nordic Cochrane Centre, The Cochrane Collaboration, 2014.) and stata14.0 (Stata/SE 14.0 for Windows (64-bit). Revision Apr 22, 2015.Copyright 1985-2015 StataCorp LP). were used for Statistical analysis. Registration number: CRD42020220570.

**Results:**

After layer-by-layer screening, 20 RCTs were finally included, which include a total of 1363 patients and 4 kinds of RCT of TCM injection (12 studies on Danhong injection, 5 studies on Ligustrazine injection, 2 studies on Huangqi injection and 1 study on Dazhu hongjingtian injection). The results showed: Clinical effective rate: Danhong Injection (Odds ratio [OR] = 3.94, 95% CI [2.34, 6.64], moderate certainty of evidence), Huangqi injection (OR = 3.40, 95% CI [1.38, 8.41], moderate certainty of evidence) and Ligustrazine injection (OR = 2.74, 95% CI [1.62, 4.64], moderate certainty of evidence) combined with conventional treatment had better curative efficacy than that of the conventional treatment group. SUCRA Ranking: Danhong (80.5) > Huangqi (68.5) > Ligustrazine (52.9) > Dazhu hongjingtian (44.3) > Conventional treatment (3.8); Forced Expiratory Volume In 1s/Forced vital capacity%: SUCRA Ranking: Danhong (80.0) > Ligustrazine (62.9) > Conventional treatment (2.1); Carbon monoxide diffusing capacity%: SUCRA Ranking: Ligustrazine (89.9) > Dazhu hongjingtian (63.4) > Danhong (44.9) > Conventional treatment (1.8); Partial pressure of Oxygen: SUCRA Ranking: Dazhu Hongjingtian (87.1) > Danhong (78.8) > Ligustrazine (34.0) > Conventional treatment (0.0); Partial pressure of carbon dioxide: SUCRA Ranking: Danhong (99.3) > Ligustrazine (50.3) > Conventional treatment (0.4). No obvious adverse reactions were found in all studies.

**Conclusion:**

The four TCM injections combined with conventional treatment can effectively improve the clinical indicators of patients with IPF, and the improvement effect of Danhong injection was more obvious.

## 1. Introduction

Idiopathic pulmonary fibrosis (IPF) is a group of diffuse lung parenchymal lesions of unknown cause. It has the characteristics of high morbidity and mortality, and its morbidity shows an increasing trend as people get older. The average survival period of patients diagnosed with IPF is 2.53 to 5 years,^[1]^ and the patient’s survival rate is significantly reduced over the time. The survival rates of 3-year’s and 5-year’ s are about 50% and 20% respectively.^[1]^ The reaction and interaction of fibroblasts and alveolar epithelial cells is the main link in the pathogenesis of pulmonary fibrosis.^[2]^ Although there is a certain understanding of its pathogenesis, the etiology and pathogenesis of the disease are very complicated, so far there is no recognized pathogenesis theory and effective treatment methods. At present, the treatment drugs for pulmonary fibrosis include glucocorticoids, glucocorticoids combined with immunosuppressant, colchicine, cyclosporine, interferon, and etanercept, but the effects of them are not ideal, and the side effects of long-term taking are obvious, thus it is difficult for patients to adhere to it. These problems have brought a heavy burden to the patients and the society.^[2]^ Pefenidone and Nintedani were recommended in the 2015 Guidelines, but due to factors such as large side effects and high prices, these two drugs are not widely used in China.^[3]^ In addition, the Guidelines also pointed out that there was no evidence that any drugs other than lung transplantation can effectively treat IPF.^[4]^Therefore, it is imperative to explore new drugs to control or treat it. At present, traditional medicines have been widely studied and used as alternative medicines in different disciplines, such as biology, immunology, chemistry, etc.^[5]^ Even some scholars believe that in the future, modern medicine will take traditional medicine as a form of alternative medicine as the main development direction.^[6]^ At present, many studies on the substitution of traditional medicines for different disease groups have been completed.^[7,8]^ Modern medicine should be closely integrated with traditional medicine and learn from each other’s strengths. As complementary medicine, traditional medicine can be implemented through an “integrative approach,” that is, individualized strategies are adopted according to the specific conditions of different patients. All appropriate interventions are taken in the series of scientific branches to restore the health of patients.^[9]^

IPF has been recorded in ancient Chinese medical literature, and most believe that it was part of the categories of “pulmonary flaccidity” and “pulmonary arthralgia.”^[10]^ In terms of pathogenesis, its essence is “qi deficiency and blood stasis,” in which qi deficiency refers to the deficiency of the lung and kidney, and blood stasis refers to the mutual obstruction of phlegm and blood stasis. Qi deficiency and Blood stasis are the most important targets for the occurrence and development of IPF. They can be considered that the pathological process of IPF has Qi deficiency and Blood stasis. Therefore, we should invigorate Qi and promote blood circulation to remove blood stasis in treating IPF.^[10]^ In China, the traditional Chinese medicine (TCM) injections widely used in the adjuvant treatment of IPF mainly include Danhong injection, Ligustrazine injection, Huangqi injection, Dazhu Hongjingtian injection, Safflower yellow sodium chloride injection and so on. They have the effect of invigorating Qi or promoting blood circulation to remove blood stasis. There are many clinical reports on these TCM injections and they proved the good clinical effects of them. However, TCM treatment has the characteristics of “individualization,” so it is difficult to formulate standard treatment details, which makes the quality of evidence of clinical efficacy of TCM less strong. In addition, there is currently no comparison of their efficacy in adjuvant treatment of IPF, which makes it difficult to promote the use of these TCM injections in clinical practice. Therefore, it is necessary to carry out rigorous and objective quality evaluation on clinical studies of different TCM injections, and the effectiveness analysis results obtained on this basis would be more convincing.

This study collected all current randomized controlled trials (RCTs) of TCM injection combined with conventional therapy in the treatment of IPF. A systematic review method was used to objectively evaluate the efficacy and safety of these TCM injections, and exploring a strong evidence-based medical basis for TCM injections as new alternative drugs to improve IPF.

## 2. Methods

This protocol is performed by following the PRISMA-P guidelines,^[11]^ and the present study has also been registered on PROSPERO (https://www.crd.york.ac.uk/prospero/), its registration number is: CRD42020220570.

### 2.1. Inclusion and exclusion criteria

#### 2.1..1. Types of research

RCTs of TCM injections combined with conventional treatment. These RCTs are either in English or Chinese, regardless of whether they use blinding or allocation concealment.

#### 2.1..2. Types of participants

All studies must comply with authoritative standards (It was formulated by the respiratory disease society of the Chinese Medical Association^[12]^ or American Thoracic Society and other societies in Japan and Europe).^[3]^ All patients included in the study need to exclude serious diseases related to other systems, and their gender, age, race and nationality are not restricted.

#### 2.1..3. Types of intervention

Experimental group: TCM injections was used on the basis of conventional treatment or control group. The dosage and method of administration of TCM injections were not limited. Control group: On the basis of conventional treatment, Hormones, N-acetylcysteine, Cyclophosphamide, Azathioprine and other drugs were used. The dosage, administration method and treatment course of the drugs were not limited. Conventional treatments included antibiotic and oxygen therapy, and other treatments.

#### 2.1..4. Outcomes

Primary outcome: Clinical effective rate: Comprehensive scoring method following the clinical physiological x-ray developed by Watters et al.^[13]^ Secondary outcomes: Lung function; The analysis of arterial blood gas; and Adverse effects.

#### 2.1..5. Exclusion criteria

Excluding TCM treatments in dosage forms such as decoctions, tablets, capsules, etc; Excluding non-randomized controlled trials, case reports, reviews, expert opinions, and animal experimental studies; and for the same research published many times by the same author, the one with the most complete information was retained.

### 2.2. Documents search strategy

Computer searched the 8 major databases: PubMed, EMbase, CENTRAL, MEDLINE, CBM, China National Knowledge Infrastructure, WanFang Database and VIP Chinese Science. Theme words and keywords were retrieved combining with literature retrospective and manual retrieval methods. The search terms are: “Pulmonary fibrosis” or “Pulmonary interstitial fibrosis” or “Idiopathic pulmonary fibrosis” or “Interstitial lung disease” or “IPF” and “traditional Chinese medicine” or “Drugs, Chinese Herbal” or “traditional Chinese herbal medicine” or “Chinese herb” and “injection.” The retrieval date is from construction to December 2020. Table 1 shows the search strategies in PubMed. In addition, Baidu Scholar and Google Scholar are also searched for related documents as supplements.

**Table 1:** Example of PubMed search strategy.

Number	Search Terms
#1	Mesh descriptor: (Idiopathic pulmonary fibrosis) explode all trees
#2	(((Pulmonary fibrosis[Title/Abstract]) OR (Pulmonary interstitial fibrosis[Title/Abstract])) OR (Interstitial lung disease[Title/Abstract])) OR (IPF[Title/Abstract])
#3	Or 1–2
#4	Mesh descriptor: (traditional Chinese medicine) explode all trees
#5	((Chinese Herbal[Title/Abstract]) OR (traditional Chinese herbal medicine[Title/Abstract])) OR (Chinese herb[Title/Abstract])
#6	Or 4–5
#7	Mesh descriptor: (injection) explode all trees
#8	6 and 7
#9	3 and 8

### 2.3. Documents screening and data extraction

According to the criteria of the inclusion and exclusion in this review, two reviewers (Xiaozheng Wu and Wen Li) independently screened these documents. The first step was to exclude documents that do not meet the criteria, and the second step was to read them completely. For documents with disagreements, they resolved it through discussion, or handed the disagreement to a third author (Yunzhi Chen) to decide whether there is a disagreement. For documents whose results are not detailed or lacking information, they sent emails as much as possible to contact the original authors for details. When extracting data, the design of the table followed the “PICOST” principle (participants, intervention, comparison, results, study design, time).

### 2.4. Quality evaluation

The standard for evaluating the quality of literature is the modified version of the Jadad scale.^[14]^

### 2.5. Bias risk assessment

The quality of this study was evaluated one by one by Cochrane 5.1.0 bias risk assessment tool, the assessment content includes: Random allocation method; Hidden grouping method; method of double blinding; Implement blind evaluation of results; Incomplete data of the results; Selective result report; and Other biases.

### 2.6. Grading of Recommendations Assessment, Development and Evaluation (GRADE) standard evaluates the results of network meta-analysis

The GRADE approach specific to network meta analysis served to assess the certainty in the evidence (quality of evidence) associated with specific comparisons, including direct, indirect, and final network meta-analysis estimates.^[15]^

Our confidence assessment addressed the RoB (in individual studies), imprecision, inconsistency (heterogeneity in estimates of effect across studies), indirectness (related to the question or due to intransitivity), and publication bias.^[15]^ Incoherence assessment was not needed in this analysis as all estimates included only direct (Interventions vs Conventional treatment) or only indirect evidence (for all other comparisons). The evaluation method in the reference^[16]^: For direct comparisons, the estimated starting point of certainty was “high,” and for indirect comparisons, the starting certainty was reduced to “moderate.” Due to the small number of studies included in the direct comparison, it was not possible to formally assess publication bias based on statistical standards. Although considering the small amount of research and for-profit interests, the possibility of this bias was real, but we believed that this concern was not enough to further reduce the certainty of the evidence.

### 2.7. Patient and public involvement

No patients or the general public involved.

### 2.8. Statistical analysis

In this study, Revman5.3 and Stata14.0 analysis software were used for statistical analysis of all data. The dichotomous variable data included in this study were statistically analyzed by using odds ratio (OR) values and 95% CI statistics. The continuous variable data were statistically analyzed by using mean difference (MD) and 95% CI statistics. And in this study, “cure,” “markedly effective” and “effective” were combined into “effective” since “cured,” “markedly effective,” “effective” and “ineffective” are the four levels of evaluation of curative effect generally recognized by the state or committee while meta-analysis is a binary variable data. When the data between groups were sufficiently similar (*P* > .1, *I*^2^ < 50%), the fixed-effects model would be used for pooled analysis. Influence analysis would be applicable when the heterogeneity originated from low-quality research. Descriptive analysis was used for data that cannot be combined. The analysis of publication bias used a funnel chart.

## 3. Results

### 3.1. Documents search results

**Figure 1.**
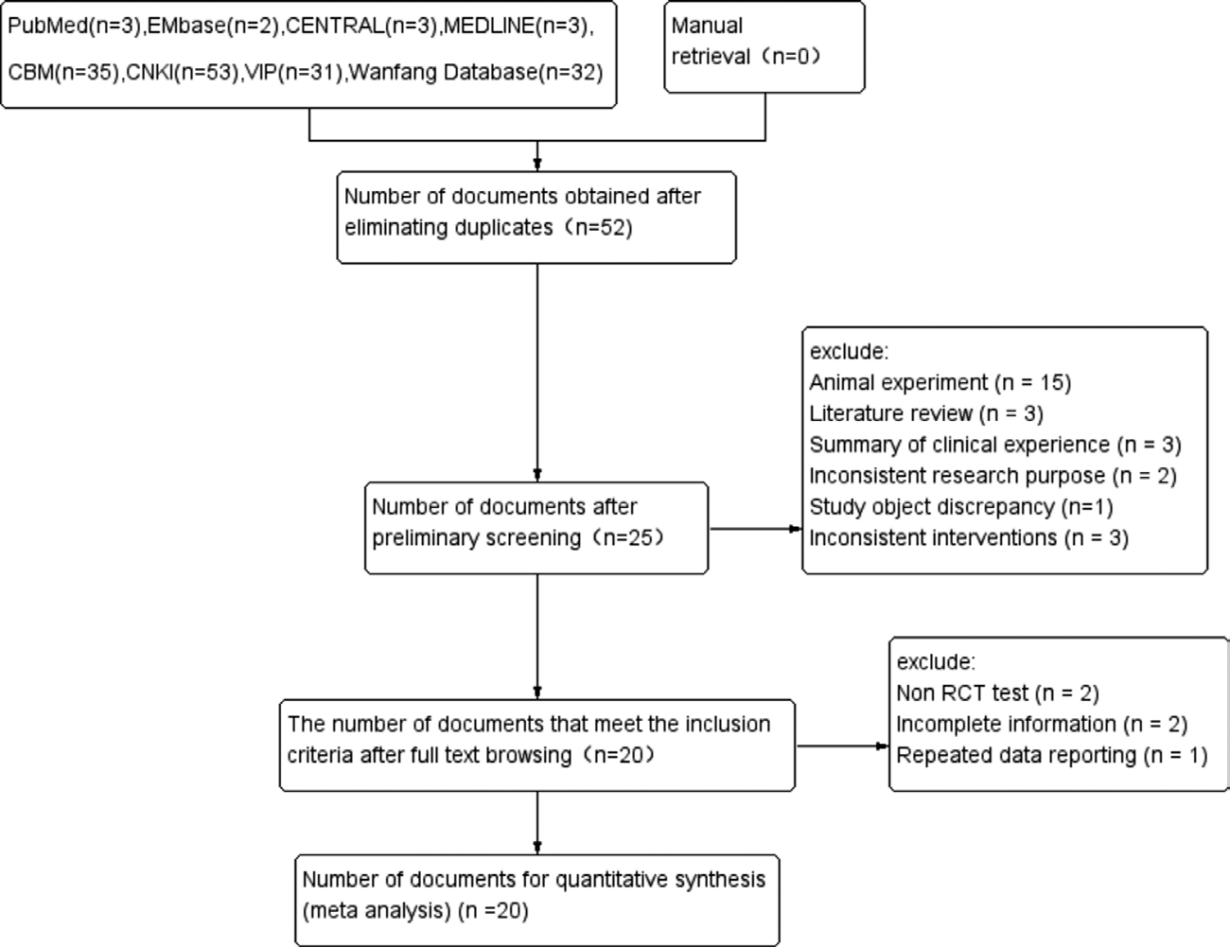
PRISMA literature screening flow chart.

In this systematic review, we initially screened 162 relevant documents from 8 databases, and after stratified screening, 20 studies were finally included.^[17–36]^ No English documents were retrieved. These included literature contained a total of 1363 patients (there were 696 cases and 667 cases in the treatment group and the control group respectively), including 4 RCTs of TCM injections (12 studies of Danhong injection,^[17,18,20,21,24,25,28–30,32,35,36]^ 5 Ligustrazine injection,^[19,23,26,27,33]^ 2 studies of Huangqi injection^[22,34]^ and 1 study of Dazhu hongjingtian injection^[31]^). A document screening flowchart, as shown in Figure 1, was made according to the PRISMA statement^[11]^ which requires that each systematic review to have. In addition, the characteristic data of the included studies are shown in Table 2.

**Table 2:** Basic features of the included study.

Studies	Sample (n)		Gender (male/female) (n)	Age (Year)	Average course of disease (Year)	Outcomes	Course (week)	Adverse reactions	Interventions	
	E	C							Experimental group	Control group
Cai 2015^[17]^	30	30	E:20/10 C:21/9	E:69.1 C:65.8	E:3.1 C:2.8	DLCO%, PaO_2_	6	E:3/30;C:3/30. There were 3 cases in the experimental group and 3 cases in the control group. After adjusting to Erythromycin Tablets, they disappeared after taking medicine after meal (more consideration of erythromycin gastrointestinal stimulation), and no obvious adverse reactions were found.	Danhong injection, Erythromycin Tablets, routine treatment	Erythromycin Tablets, routine treatment
Chen 2014^[18]^	45	45	E:27/18 C:25/20	E:50.2 ± 9.6 C:49.5 ± 8.7	E:4.8 ± 2.1 C:4.6 ± 1.8	PaO_2_	12	Not described	Danhong injection, Prednisolone , routine treatment	Prednisolone, routine treatment
Huang 2010^[19]^	30	30	E:15/15 C:14/16	E: 50~70 C: 52~71	E: 5 mo~4.5 yr C: 5 mo~5 yr	Clinical efficiency, FEV_1_/FVC%, DLCO%	12	Not described	Ligustrazine injection, routine treatment	Prednisone, routine treatment
Li 2012^[20]^	34	34	E:11/13 C:14/10	E:56.32 ± 3.29 C:56.38 ± 3.27	–	Clinical efficiency, PaO_2_	8	Not described	Danhong injection, Prednisone, routine treatment	Prednisolone, routine treatment
Lin 2016^[21]^	35	35	E:20/15 C:20/15	E:62. 41 ± 9. 82 C:61.53 ± 12.52	E:2.54 ± 1.71 C:2.85 ± 1.62	FEV_1_/FVC%, PaO_2_	12	Non	Danhong injection, Methylprednisolone, routine treatment	Methylprednisolone, routine treatment
Liu 2012^[22]^	23	23	29/17	40~68	–	Clinical efficiency	12	Non	Huangqi injection, Budesonide, routine treatment	Budesonide, routine treatment
Liu 2015^[23]^	35	35	E:29/6 C:28/7	E: 55.8 ± 12.9 C: 53.6 ± 11.7	–	Clinical efficiency, DLCO%, PaO_2_, PaCO_2_	12	E:7/35 (edema in 2 cases, hypertension in 2 cases, gastrointestinal bleeding in 2 cases and depression in 1 case) C:7/35 (edema in 3 cases, hypertension in 1 case, hyperglycemia in 1 case, oral infection in 1 case, and bone pain in 1 case)	Ligustrazine injection, budesonide, routine treatment	Budesonide, routine treatment
Ren 2012^[24]^	24	24	–	E:50.63 ± 10.63 C:50.86 ± 10.22	E:6.07 ± 3.34 C:6.36 ± 3.22	Clinical efficiency, PaO_2_	6	Not described	Danhong injection, Prednisolone, routine treatment	Prednisolone, routine treatment
Sun 2015^[25]^	35	32	E:19/16 C:18/14	E:48.2 ± 16.3 C:50.8 ± 17.4	E:4.6 ± 2.8 C:4.6 ± 2.7	Clinical efficiency, PaO2	12	Not described	Danhong injection, Edaravone, routine treatment	Edaravone, routine treatment
Wang 2007^[26]^	56	30	E:36/20 C:18/12	26~58	2 mo–1 yr	Clinical efficiency	8	Not described	Ligustrazine injection, routine treatment	Prednisone, routine treatment
Wang 2013^[27]^	16	16	16/16	42~70	3~8	Clinical efficiency, FEV_1_/FVC%, PaO_2_, PaCO_2_	2	Not described	Ligustrazine injection, prednisone, routine treatment	Prednisone, routine treatment
Wang 2016^[28]^	25	25	E:14/11 C:13/12	E: 36–58 C:36–67	–	Clinical efficiency, PaO2	6	Not described	Danhong injection, Prednisolone, routine treatment	Prednisolone, routine treatment
Wang 2020^[29]^	60	60	E:28/32 C:26/34	E: 64.0 ± 5.7 C:63.0 ± 6.2	E:6.3 ± 1.8 C:5.9 ± 2.7	DLCO%, PaO_2_, PaCO2	12	Not described	Danhong injection, Acetylcysteine, routine treatment	Acetylcysteine, routine treatment
Wu 2018^[30]^	30	30	E:18/12 C:19/11	E:61.56 ± 12.33 C:61.80 ± 12.18	E:2.88 ± 1.56 C:2.52 ± 1.60	PaO_2_	12	Non	Danhong injection, Methylprednisolone, routine treatment	Methylprednisolone, routine treatment
Yang 2014^[31]^	20	20	–	18~65	–	Clinical efficiency, DLCO%, PaO_2_	6	Non	Dazhu Hongjingtian injection, routine treatment	Prednisone, routine treatment
Yin 2011^[32]^	41	41	E:20/21 C:19/22	E: 52 C: 55	–	Clinical efficiency, PaO2	6	Not described	Danhong injection, Prednisolone, routine treatment	Prednisolone, routine treatment
Yu 2016^[33]^	25	24	27/22	55~82	3–18 mo	Clinical efficiency, DLCO%, PaO_2_	4	Not described	Ligustrazine injection, methylprednisolone, routine treatment	Methylprednisolone, routine treatment
Yuan 2020^[34]^	42	42	E:25/17 C:26/16	E:60.42 ± 6.59 C:60.18 ± 6.43	E:5.24 ± 1.87 C:5.21 ± 1.75	Clinical efficiency	2	Not described	Huangqi injection, Budesonide, routine treatment	Budesonide, routine treatment
Zhao 2016^[35]^	40	40	E:26/14 C:27/13	E:62.3 ± 8.9 C:62.8 ± 8.7	E:2.8 ± 1.6 C:2.8 ± 1.5	FEV_1_/FVC%, PaO_2_	12	Not described	Danhong injection, Acetylcysteine, routine treatment	Acetylcysteine, routine treatment
Zhou 2012^[36]^	50	51	E:28/22 C:27/24	E:55.31 ± 9.36 C:54.96 ± 9.72	E:4 mo–2 yr C:3 mo–2 yr	Clinical efficiency	8	Not described	Danhong injection, Prednisone, Telmisartan, routine treatment	Prednisone, Telmisartan, routine treatment

Note: C = control group, E = experimental group, mo = month; yr = year.

DLCO% = Carbon monoxide diffusing capacity%, FEV_1_/FVC% = Forced Expiratory Volume In 1s/Forced vital capacity%, PaCO2 = partial pressure of carbon dioxide, PaO_2_ = partial pressure of oxygen.

### 3.2. Quality assessment

A total of 20 RCTs conducted in China were included in this systematic review. Their data was complete, and there was no data loss. They all mentioned the use of randomization, described the comparability of baseline data, the treatment measures and efficacy outcomes of the two groups in detail. However, they did not clarify the specific allocation concealment methods or blinding methods. The results showed that 5 articles were 3 points^[17,18,23,25,30]^ and 15 articles were 2 points. In general, 15 papers were low-quality studies, and 5 were medium-quality studies. The evaluation results of the Jadad scale^[14]^ are shown in Table 3.

**Table 3:** Quality evaluation of original research.

Studies	Randomization method	Allocation concealment	Blind method	Loss to follow-up	Baseline comparability	Jadad score
Cai 2015^[17]^	Random	Not described	Not described	Adverse reactions	No significant difference	3
Chen 2014^[18]^	Random number table method	Not described	Not described	Not described	No significant difference	3
Huang 2010^[19]^	Random	Not described	Not described	Not described	No significant difference	2
Li 2012^[20]^	Random	Not described	Not described	Not described	No significant difference	2
Lin 2016^[21]^	Random	Not described	Not described	Not described	No significant difference	2
Liu 2012^[22]^	Random	Not described	Not described	Not described	No significant difference	2
Liu 2015^[23]^	Random	Not described	Not described	Adverse reactions	No significant difference	3
Ren 2012^[24]^	Random	Not described	Not described	Not described	No significant difference	2
Sun 2015^[25]^	Random number table method	Not described	Not described	Not described	No significant difference	3
Wang 2007^[26]^	Random	Not described	Not described	Not described	No significant difference	2
Wang 2013^[27]^	Random	Not described	Not described	Not described	No significant difference	2
Wang 2016^[28]^	Random	Not described	Not described	Not described	No significant difference	2
Wang 2020^[29]^	Random	Not described	Not described	Not described	No significant difference	2
Wu 2018^[30]^	Random number table method	Not described	Not described	Not described	No significant difference	3
Yang 2014^[31]^	Random	Not described	Not described	Not described	No significant difference	2
Yin 2011^[32]^	Random	Not described	Not described	Not described	No significant difference	2
Yu 2016^[33]^	Random	Not described	Not described	Not described	No significant difference	2
Yuan 2020^[34]^	Random	Not described	Not described	Not described	No significant difference	2
Zhao 2016^[35]^	Random	Not described	Not described	Not described	No significant difference	2
Zhou 2012^[36]^	Random	Not described	Not described	Not described	No significant difference	2

### 3.3. Evaluate the original literatures

**Figure 2.**
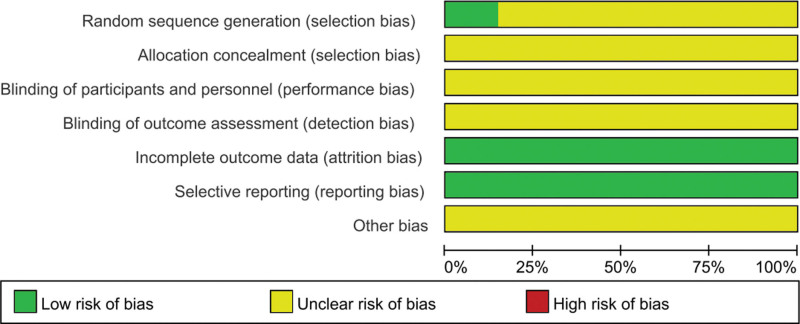
Risk of bias graph.

**Figure 3.**
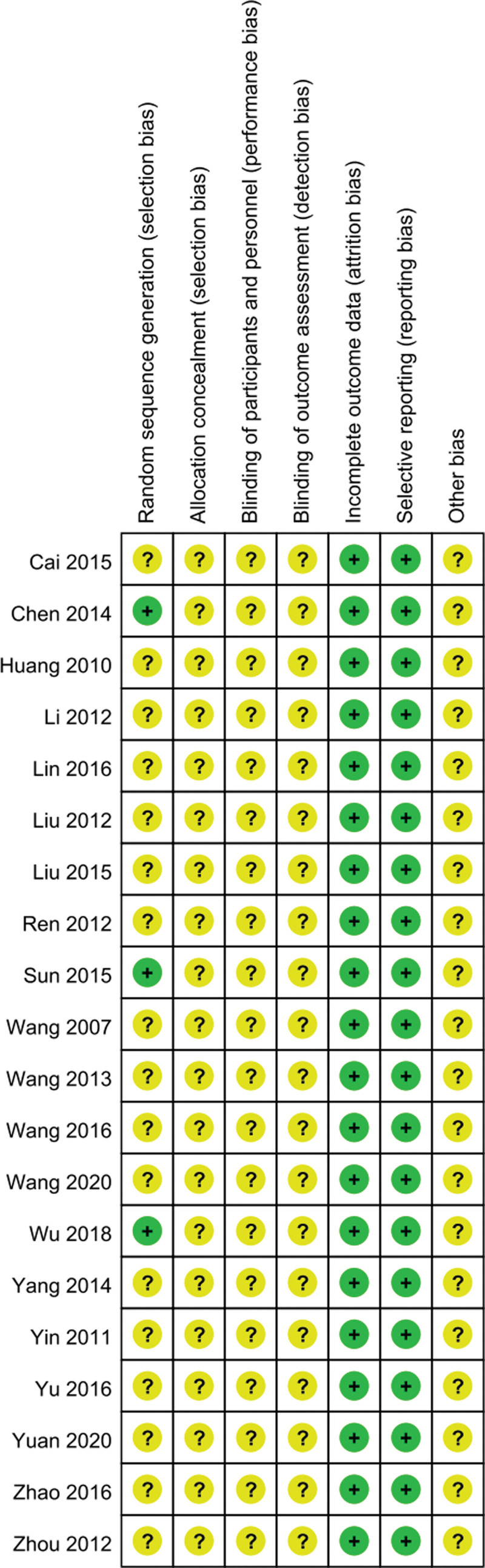
Risk of bias summary chart.

The results of Figures 2 and 3 suggested that the low-risk and medium-risk generated by random sequences account for 31% and 69% of the original research selection bias respectively. Therefore, there are certain biases in the literatures, such as selection, implementation and measurement biases.

### 3.4. The results of network meta-analysis

#### 3.4..1. Clinical effective rate

**Figure 4.**
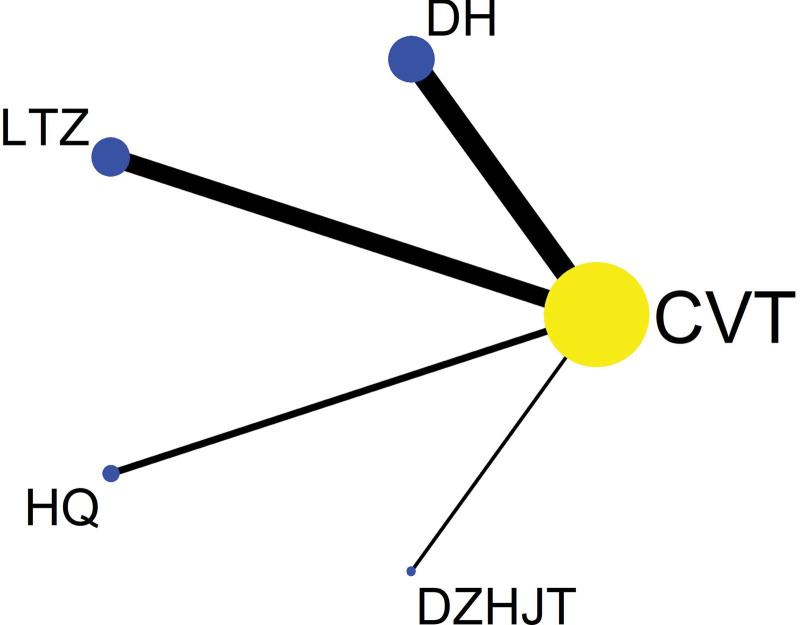
Network evidence map of network meta analysis.

**Figure 5.**
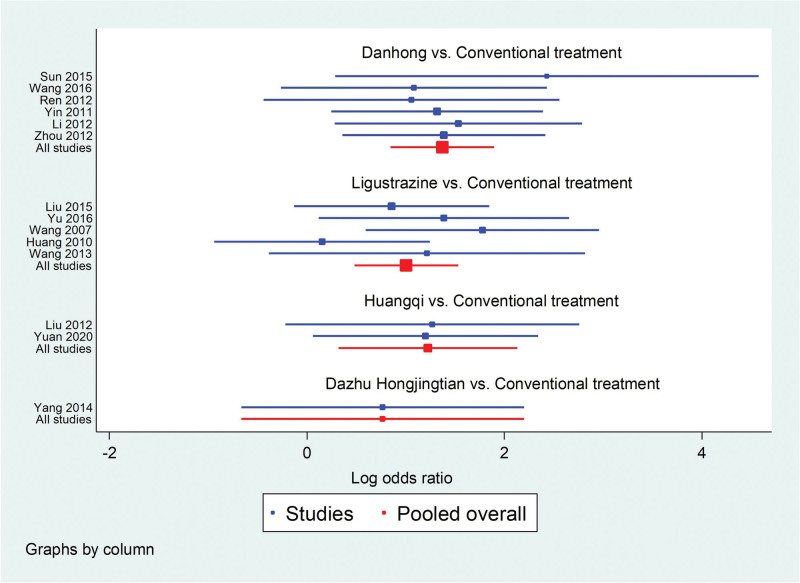
Consistency test forest map.

Among the 20 literatures included, 14 reported the effective rate of TCM, of which Danhong injection treated IPF in 6 articles,^[20,24,25,28,32,36]^ Ligustrazine injection treatment in 5 articles,^[19,23,26,27,33]^ Huangqi injection treatment in 2 articles,^[22,34]^ and Dazhu Hongjingtian injection in 1 article,^[31]^ as shown in Figure 4. Statistical analysis was carried out with OR as effect size and 95% confidence interval (CI). Stata/SE 14.0 was used to test the heterogeneity of clinical efficiency. The results showed that *I*^2^ = 0%, *P* = .90, and the heterogeneity test met the criteria of *I*^2^ ≤ 50% and *P* ≥ .05, and the effect size could be combined for Meta-analysis. Consistency model results showed that all study effects (OR) > 0, indicated that the consistency of the results is credible, as shown in the Figure 5.

**Figure 6.**
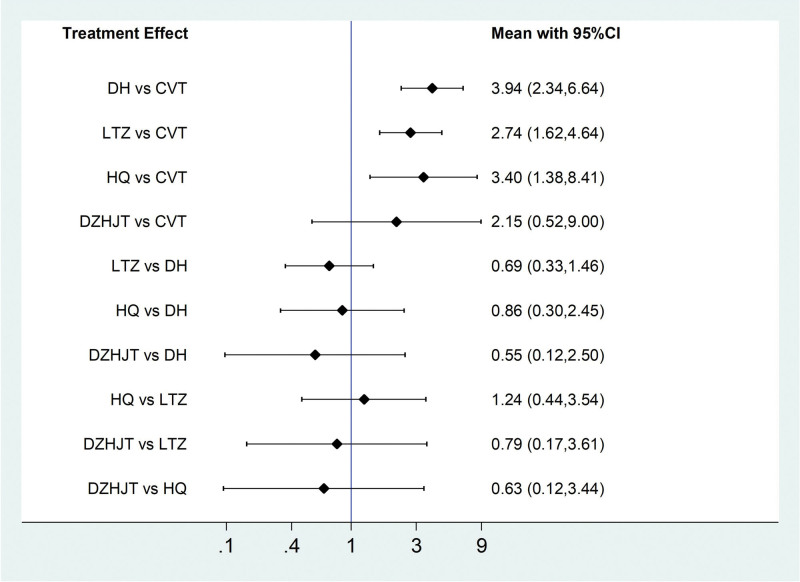
Comparative forest chart of clinical effective rate.

**Figure 7.**
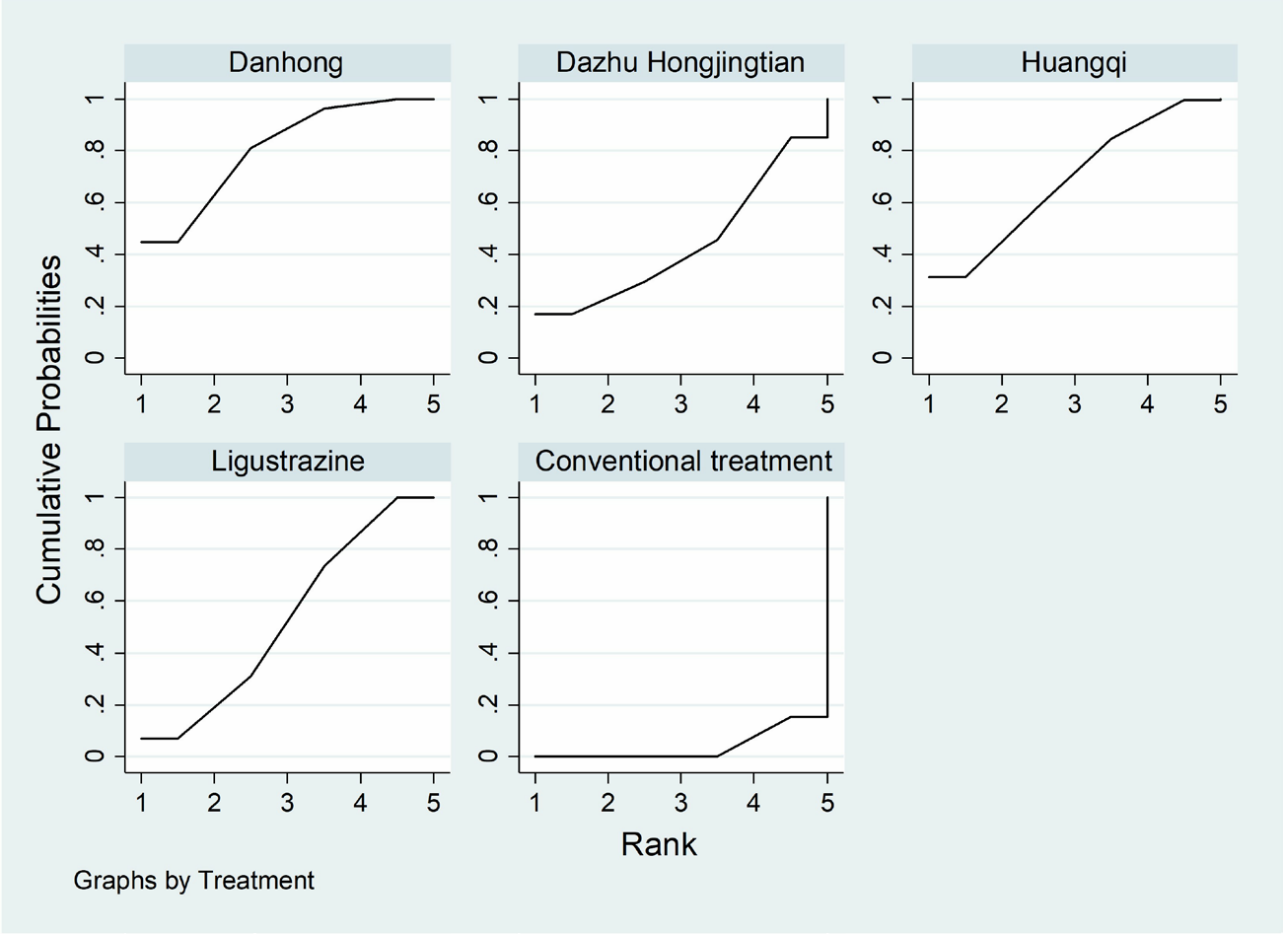
SUCRA ranking chart of clinical effective rate.

According to the results of network meta-analysis with Stata14.0 software, the effective rates of Danhong injection (OR = 3.94, 95% CI [2.34, 6.64], moderate certainty of evidence), Huangqi injection (OR = 3.40, 95% CI [1.38, 8.41], moderate certainty of evidence) and Ligustrazine injection (OR = 2.74, 95% CI [1.62, 4.64], moderate certainty of evidence) were all better than those of the conventional treatment group, and the differences were statistically significant. There was no difference in the effective rate between Dazhu hongjingtian injection (OR = 2.15, 95% CI [0.52, 9.00], low certainty of evidence) and the conventional treatment group. And there was no difference in the effective rate between the four traditional Chinese medicine injections. From the SUCRA ranking order, Danhong (80.5) > Huangqi (68.5) > Ligustrazine (52.9) > Dazhu hongjingtian (44.3) > Conventional treatment (3.8). See Figures 6 and 7 and Tables 4 and 5 for details.

**Table 4:** NMA on clinical effective rate.

Danhong	0.86 (0.30, 2.45)	0.69 (0.33, 1.46)	0.55 (0.12, 2.50)	0.25 (0.15, 0.43)
	⊕⊝⊝⊝	⊕⊕⊝⊝	⊕⊝⊝⊝	⊕⊕⊕⊝
	very low^2^^,^^3^	low^1^^,^^4^	very low^3^^,^^4^	moderate^1^
1.16 (0.41,3.29)	Huangqi	0.80 (0.28, 2.29)	0.63 (0.12, 3.44)	0.29 (0.12, 0.73)
⊕⊝⊝⊝		⊕⊝⊝⊝	⊕⊕⊝⊝	⊕⊕⊕⊝
Very low^2^^,^^3^		Very low^2^^,^^3^	Low^1^^,^^4^	Moderate^1^
1.44 (0.69, 3.02)	1.24 (0.44, 3.54)	Ligustrazine	0.79 (0.17, 3.61)	0.37 (0.22, 0.62)
⊕⊕⊝⊝	⊕⊝⊝⊝		⊕⊕⊝⊝	⊕⊕⊕⊕
Low^1^^,^^4^	Very low^2^^,^^3^		Low^1^^,^^2^	Moderate^2^
1.83 (0.40, 8.39)	1.58 (0.29, 8.58)	1.27 (0.28, 5.83)	Dazhu Hongjingtian	0.46 (0.11, 1.94)
⊕⊝⊝⊝	⊕⊕⊝⊝	⊕⊕⊝⊝		⊕⊕⊝⊝
Very low^3^^,^^4^	Low^1^^,^^4^	Low^1^^,^^2^		Low^3^
**3.94 (2.34, 6.64**)	**3.40 (1.38, 8.41**)	**2.74 (1.62, 4.64**)	2.15 (0.52, 9.00)	Conventional
⊕⊕⊕⊝	⊕⊕⊕⊝	⊕⊕⊕⊕	⊕⊕⊝⊝	
Moderate^1^	Moderate^1^	Moderate^2^	Low^3^	

Note:

1 Certainty lowered for imprecision.

2 Certainty lowered for individual study risk of bias.

3 Certainty lowered two levels for imprecision.

4 Certainty lowered for indirectness.

GRADE Working Group grades of evidence – High quality: Further research is very unlikely to change our confidence in the estimate of effect; Moderate quality: Further research is likely to have an important impact on our confidence in the estimate of effect and may change the estimate; Low quality: Further research is very likely to have an important impact on our confidence in the estimate of effect and is likely to change the estimate; Very low quality: We are very uncertain about the estimate.

NMA = network meta analysis.

**Table 5:** SUCRA value of clinical effective rate.

Treatment	SUCRA	PrBest	MeanRank
Conventional treatment	3.8	0.0	4.8
Danhong	80.5	44.7	1.8
Ligustrazine	52.9	7.1	2.9
Huangqi	68.5	31.4	2.3
Dazhu Hongjingtian	44.3	16.8	3.2

#### 3.4..2. Pulmonary function

##### 3.4..2..1. Forced Expiratory Volume In 1s/Forced vital capacity% (FEV_1_/FVC%)

**Figure 8.**
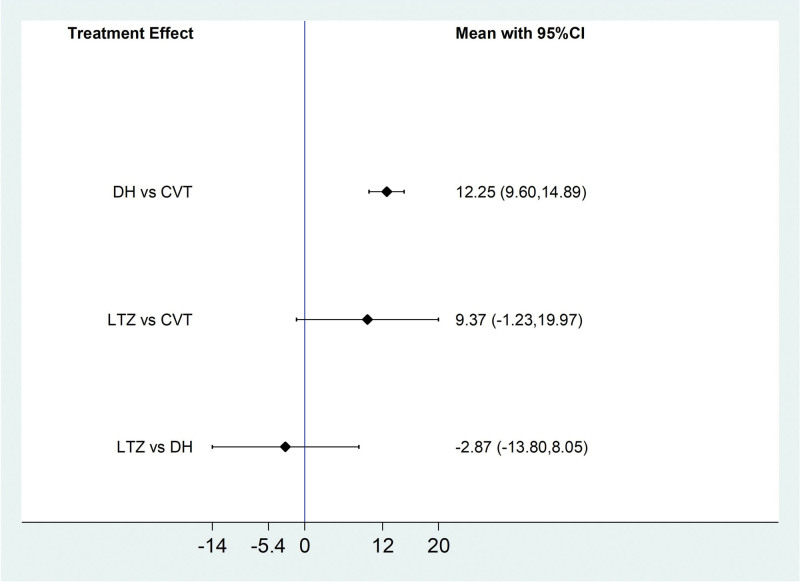
Comparative forest chart of FEV_1_/FVC%. FEV1/FVC% = Forced Expiratory Volume In 1s/Forced vital capacity%.

**Figure 9.**
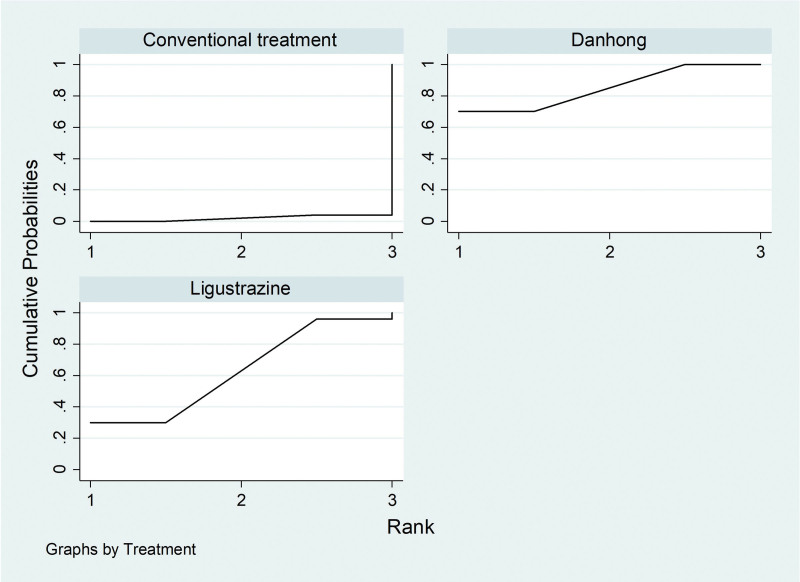
SUCRA ranking chart of FEV_1_/FVC%. FEV_1_/FVC% = Forced Expiratory Volume In 1s/Forced vital capacity%.

Among the 20 literatures, a total of 4 literatures reported the FEV_1_/FVC% of IPF patients treated with Traditional Chinese medicine injection, including 2 (Danhong injection) for IPF^[21,35]^ and 2 (Ligustrazine injection) for IPF.^[19,27]^According to the network meta-analysis results of Stata14.0 software, Danhong injection (MD = 12.25, 95% CI [9.60, 14.89], moderate certainty of evidence) improved FEV_1_/FVC% better compared with conventional treatment group, and the difference was statistically significant. Ligustrazine injection (MD = 9.37, 95% CI [−1.23, 19.97], low certainty of evidence) showed no difference compared with the conventional treatment. There was no difference between Danhong injection and Ligustrazine injection. From the SUCRA ranking order, danhong (80.0) > Ligustrazine (62.9) > Conventional treatment (2.1). See Tables 6 and 7 and Figures 8 and 9 for details.

**Table 6:** NMA on FEV_1_/FVC%.

Danhong	−2.87 (−13.80, 8.05)	−12.25 (−14.89, −9.60)
	⊕⊕⊝⊝	⊕⊕⊕⊝
	low^3^	moderate^2^
2.87 (−8.05, 13.80)	Ligustrazine	−9.37 (−19.97, 1.23)
⊕⊕⊝⊝		⊕⊕⊝⊝
Low^3^		Low^1^^,^^4^
**12.25 (9.60, 14.89**)	9.37 (−1.23, 19.97)	Conventional
⊕⊕⊕⊝	⊕⊕⊝⊝	
Moderate^2^	Low^1^^,^^4^	

Note:

1 Certainty lowered for imprecision.

2 Certainty lowered for individual study risk of bias.

3 Certainty lowered two levels for imprecision.

4 Certainty lowered for indirectness.

GRADE Working Group grades of evidence – High quality: Further research is very unlikely to change our confidence in the estimate of effect; Moderate quality: Further research is likely to have an important impact on our confidence in the estimate of effect and may change the estimate; Low quality: Further research is very likely to have an important impact on our confidence in the estimate of effect and is likely to change the estimate; Very low quality: We are very uncertain about the estimate.

NMA = network meta analysis, FEV1/FVC% = Forced Expiratory Volume In 1s/Forced vital capacity%.

**Table 7:** SUCRA value of FEV_1_/FVC%.

Treatment	SUCRA	PrBest	MeanRank
Conventional treatment	2.1	0.0	3.0
Danhong	85.0	70.1	1.3
Ligustrazine	62.9	29.9	1.7

FEV_1_/FVC% = Forced Expiratory Volume In 1s/Forced vital capacity%.

##### 3.4..2..2. Carbon monoxide diffusing capacity% (DLCO%)

Of the 20 literatures included, a total of 6 literatures reported the situation of DLCO% of IPF patients treated by Traditional Chinese medicine injection, including 2 (Danhong injection),^[17,29]^3 (Ligustrazine injection)^[19,23,33]^ and 1 (Dazhu Hongjingtian injection).^[31]^According to the network meta-analysis results of Stata14.0 software, Danhong injection (MD = 5.01, 95% CI [3.81, 6.21], moderate certainty of evidence) and Ligustrazine injection (MD = 9.12, 95% CI [5.70, 12.55], very low certainty of evidence) improved DLCO% better than the conventional treatment group, and the difference was statistically significant. There was no difference in the improvement of DLCO% by Dazhu Hongjingtian injection (MD = 6.70, 95% CI [−1.06, 14.46], moderate certainty of evidence) compared with the conventional treatment group. Ligustrazine injection (MD = 4.11, 95% CI [0.49, 7.74], very low certainty of evidence) had better efficacy than Danhong injection. From the SUCRA ranking order, ligustrazine (89.9).

**Figure 10.**
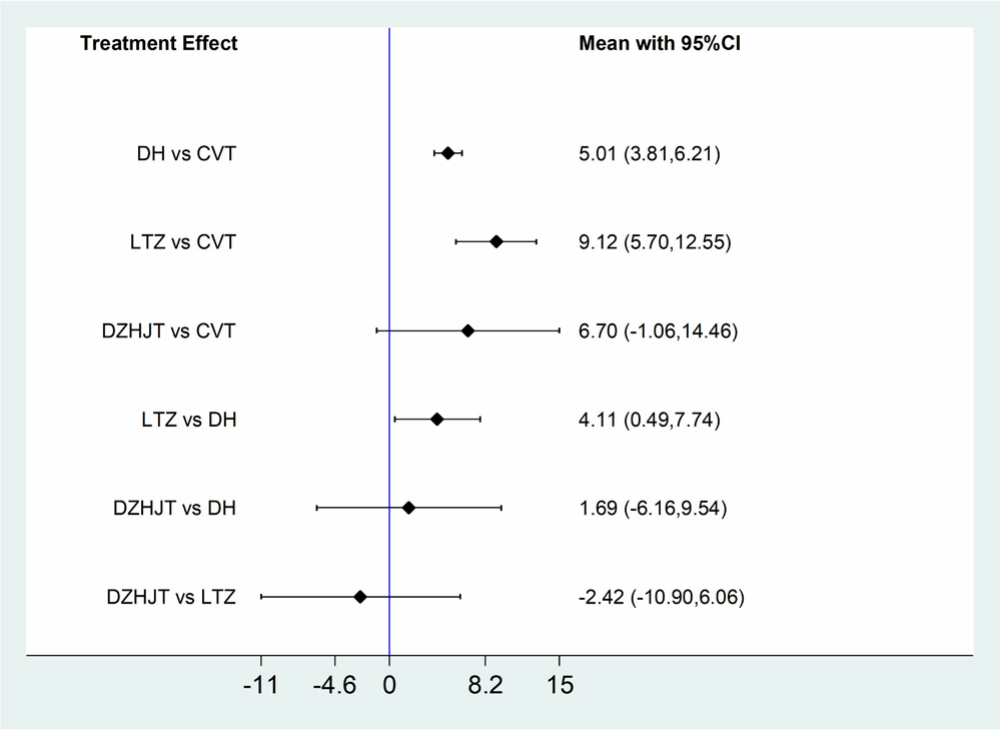
Comparative forest chart of DLCO%. DLCO% = Carbon monoxide diffusing capacity%.

**Figure 11.**
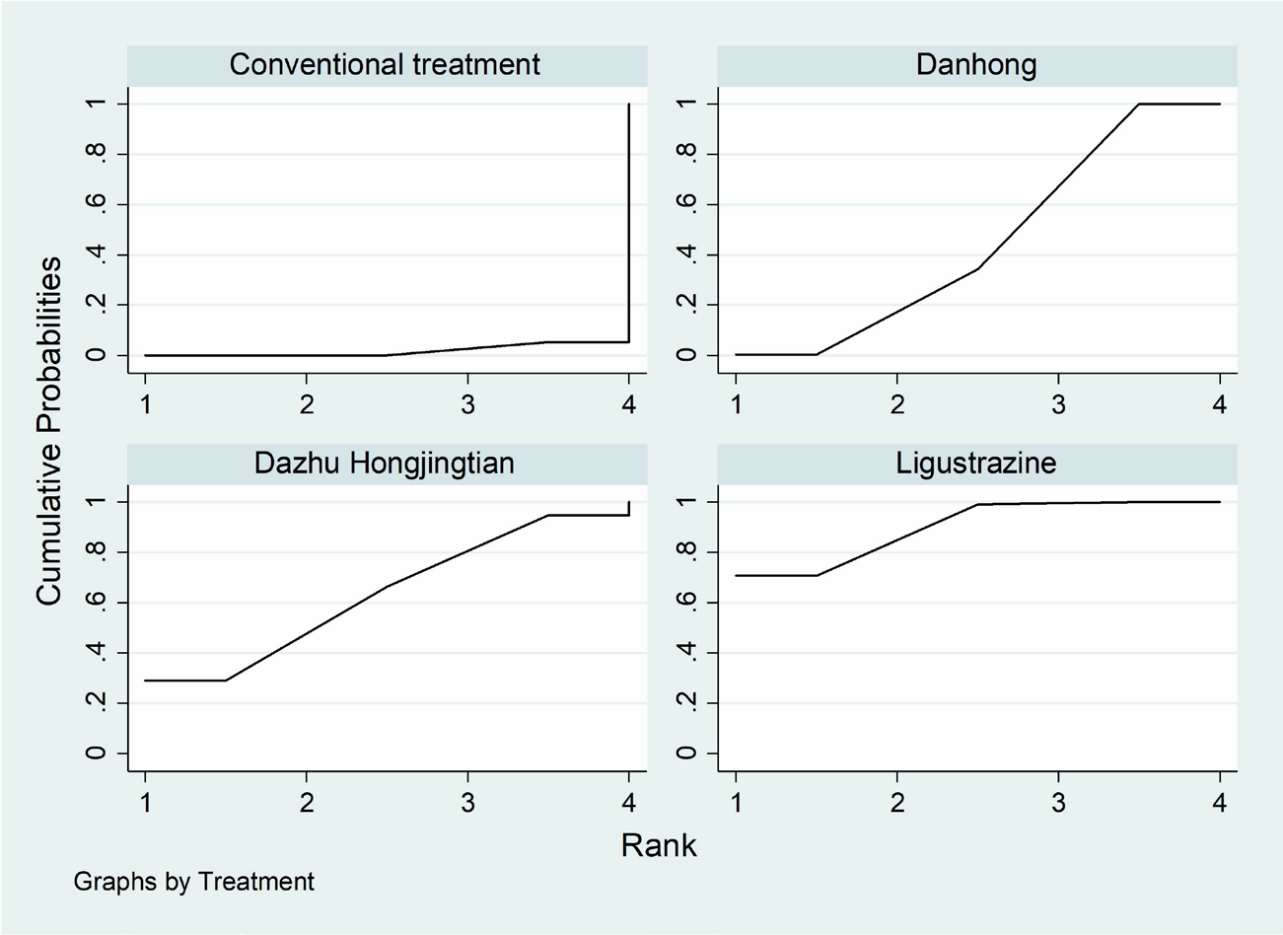
SUCRA ranking chart of DLCO%. DLCO% = Carbon monoxide diffusing capacity%.

Dazhu Hongjingtian (63.4) > Danhong (44.9) > Conventional treatment (1.8). See Tables 8 and 9 and Figures 10 and 11 for details.

**Table 8:** NMA on DLCO%.

Ligustrazine	−2.42 (−10.90, 6.06)	−4.11 (−7.74, −0.49)	−9.12 (−12.55, −5.70)
	⊕⊝⊝⊝	⊕⊝⊝⊝	⊕⊕⊕⊝
	Very low^3^^,^^4^	Very low^1^^,^^2^^,^^4^	Moderate^2^
2.42 (−6.06, 10.90)	Dazhu Hongjingtian	−1.69 (−9.54, 6.16)	−6.70 (−14.46, 1.06)
⊕⊝⊝⊝			⊕⊕⊕⊝
Very low^3^^,^^4^			Moderate^1^
**4.11 (0.49, 7.74**)	1.69 (−6.16, 9.54)	Danhong	−5.01 (−6.21, −3.81)
⊕⊝⊝⊝	⊕⊕⊝⊝		⊕⊕⊕⊝
Very low^1^^,^^2^^,^^4^	Low^2^^,^^4^		Moderate^2^
**9.12 (5.70, 12.55**)	6.70 (−1.06, 14.46)	**5.01 (3.81, 6.21**)	Conventional
⊕⊕⊕⊝	⊕⊕⊕⊝	⊕⊕⊕⊝	
Moderate^2^	Moderate^1^	Moderate^2^	

Note:

1 Certainty lowered for imprecision.

2 Certainty lowered for individual study risk of bias.

3 Certainty lowered two levels for imprecision.

4 Certainty lowered for indirectness.

GRADE Working Group grades of evidence – High quality: Further research is very unlikely to change our confidence in the estimate of effect; Moderate quality: Further research is likely to have an important impact on our confidence in the estimate of effect and may change the estimate; Low quality: Further research is very likely to have an important impact on our confidence in the estimate of effect and is likely to change the estimate; Very low quality: We are very uncertain about the estimate.

DLCO% = Carbon monoxide diffusing capacity%, NMA = network meta analysis.

**Table 9:** SUCRA value of DLCO%.

Treatment	SUCRA	PrBest	MeanRank
Conventional treatment	1.8	0.0	3.9
Danhong	44.9	0.4	2.7
Ligustrazine	89.9	70.6	1.3
Dazhu Hongjingtian	63.4	29.0	2.1

DLCO% = Carbon monoxide diffusing capacity.

#### 3.4..3. Blood gas analysis

##### 3.4..3..1. Partial pressure of Oxygen (PaO_2_)

**Figure 12.**
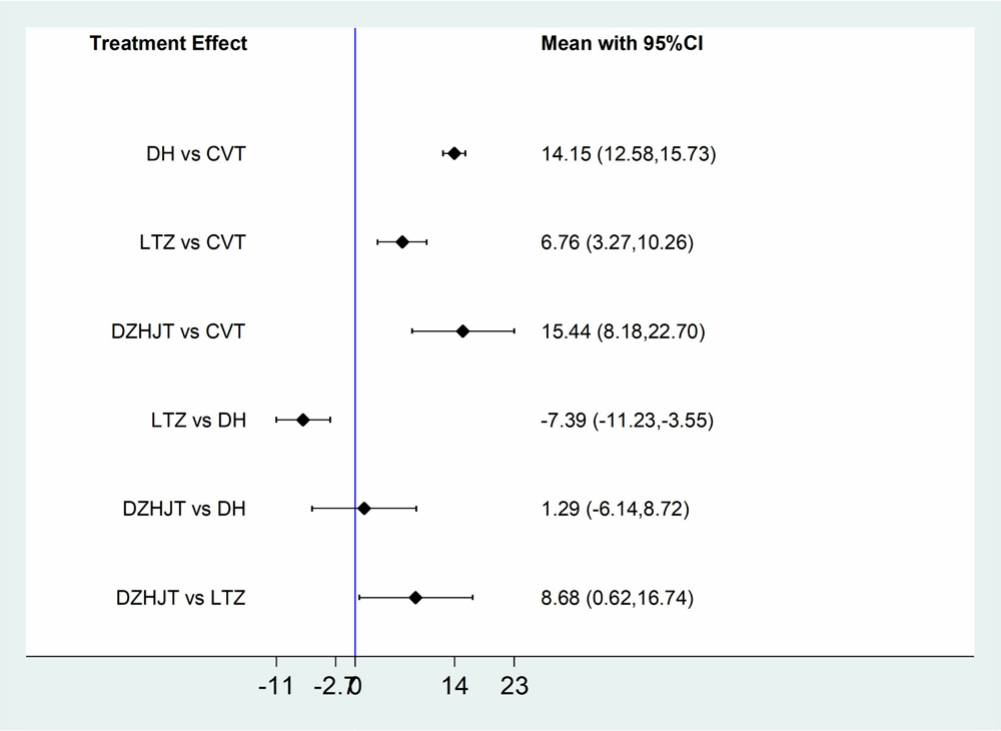
Comparative forest chart of PaO_2_. PaO_2_ = Partial Pressure of Oxygen.

**Figure 13.**
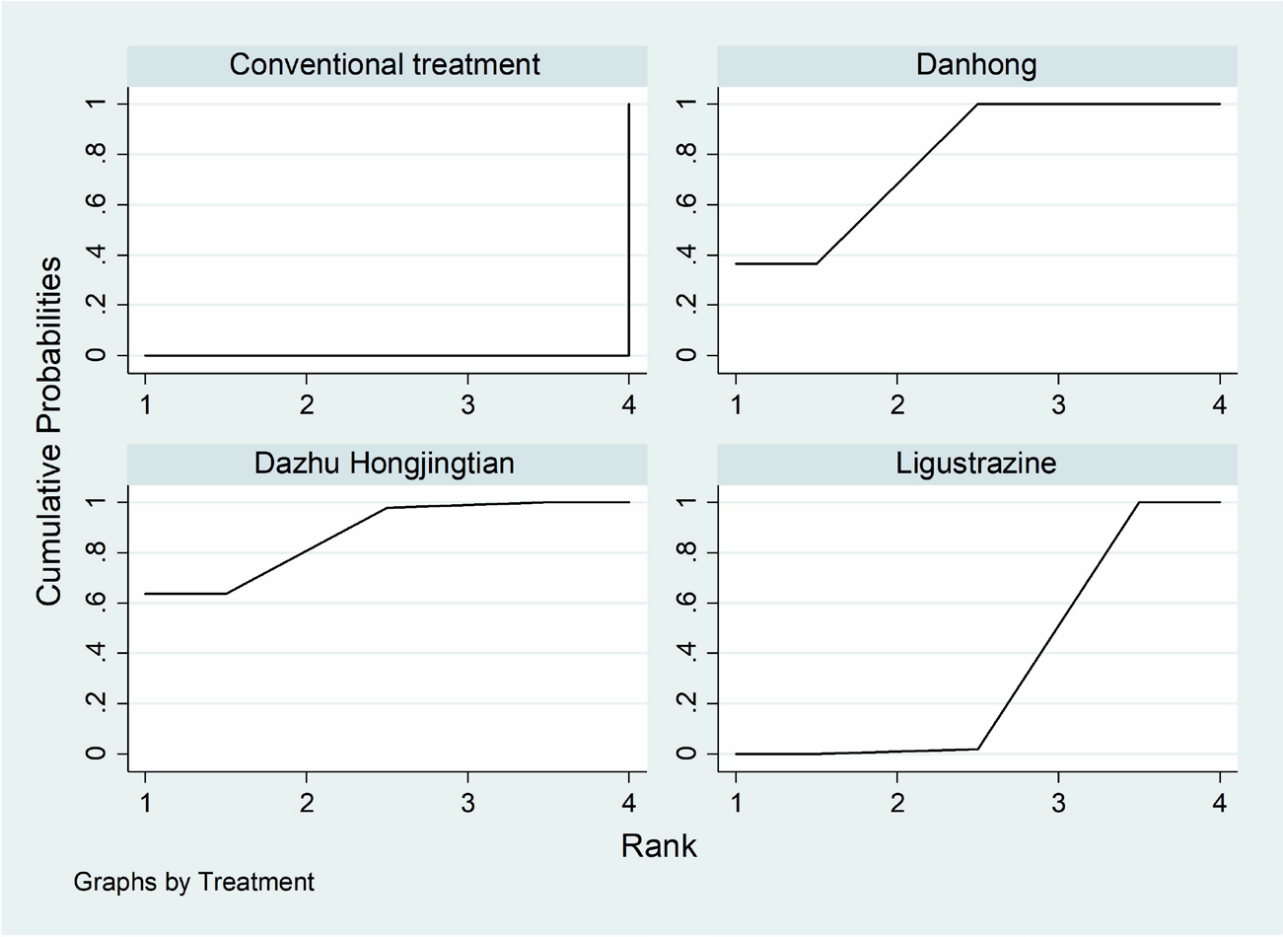
SUCRA ranking chart of PaO_2_. PaO_2_ = Partial Pressure of Oxygen.

For the 20 included literatures, a total of 15 literatures reported the PaO_2_ situation of IPF patients treated by Traditional Chinese medicine injection, among which Danhong injection treated IPF 11 articles,^[17,18,20,21,24,25,28–30,32,35]^ Ligustrazine injection treated IPF 3 articles,^[23,27,33]^ and Dazhu Hongjingtian injection treated IPF 1 article.^[31]^According to the results of network meta-analysis with Stata14.0 software, the improvement of PaO_2_ by Danhong injection (MD = 14.15, 95% CI [12.58, 15.73], moderate certainty of evidence), Ligustrazine injection (MD = 6.76, 95% CI [3.27, 10.26], moderate certainty of evidence) and Dazhu Hongjingtian injection (MD = 15.44, 95% CI [8.18, 22.70], moderate certainty of evidence) was better than that by the conventional treatment group, and this is with statistically significant differences; Danhong injection (MD = 7.39, 95% CI [3.55, 11.23], moderate certainty of evidence) and Dazhu Hongjingtian injection (MD = 8.68, 95% CI [0.62, 16.74], very low certainty of evidence) showed better efficacy than Ligustrazine injection. There was no difference between Danhong injection and Dazhu Hongjingtian injection. According to the ranking order of SUCRA, Dazhu Hongjingtian > Danhong (78.8) > Ligustrazine (34.0) > Conventional treatment (0.0). See Tables 10 and 11 and Figures 12 and 13 for details.

**Table 10:** NMA on PaO_2_.

Danhong	1.29 (−6.14, 8.72)	−7.39 (−11.23, −3.55)	−14.15 (−15.73, −12.58)
	⊕⊕⊝⊝	⊕⊕⊕⊝	⊕⊕⊕⊝
	Low^1^^,^^4^	Moderate^2^	Moderate^1^
−1.29 (−8.72, 6.14)	Dazhu Hongjingtian	−8.68 (−16.74, −0.62)	−15.44 (−22.70, −8.18)
⊕⊕⊝⊝		⊕⊝⊝⊝	⊕⊕⊕⊝
Low^1^^,^^4^		Very low^3^^,^^4^	Moderate^1^
**7.39 (3.55, 11.23**)	**8.68 (0.62, 16.74**)	Ligustrazine	−6.76 (−10.26, −3.27)
⊕⊕⊕⊝	⊕⊝⊝⊝		⊕⊕⊕⊝
Moderate^2^	Very low^3^^,^^4^		Moderate^4^
**14.15 (12.58, 15.73**)	**15.44 (8.18, 22.70**)	**6.76 (3.27, 10.26**)	Conventional
⊕⊕⊕⊝	⊕⊕⊕⊝	⊕⊕⊕⊝	
Moderate^1^	Moderate^1^	Moderate^4^	

Note:

1 Certainty lowered for imprecision.

2 Certainty lowered for individual study risk of bias.

3 Certainty lowered two levels for imprecision.

4 Certainty lowered for indirectness.

GRADE Working Group grades of evidence – High quality: Further research is very unlikely to change our confidence in the estimate of effect; Moderate quality: Further research is likely to have an important impact on our confidence in the estimate of effect and may change the estimate; Low quality: Further research is very likely to have an important impact on our confidence in the estimate of effect and is likely to change the estimate; Very low quality: We are very uncertain about the estimate.

NMA = network meta analysis, PaO_2_ = partial pressure of oxygen.

**Table 11:** SUCRA value of PaO_2_.

Treatment	SUCRA	PrBest	MeanRank
Conventional treatment	0.0	0.0	4.0
Danhong	78.8	36.5	1.6
Ligustrazine	34.0	0.0	3.0
Dazhu Hongjingtian	87.1	63.5	1.4

PaO_2_ = partial pressure of oxygen.

##### 3.4..3..2. Partial pressure of carbon dioxide (PaCO_2_)

**Figure 14.**
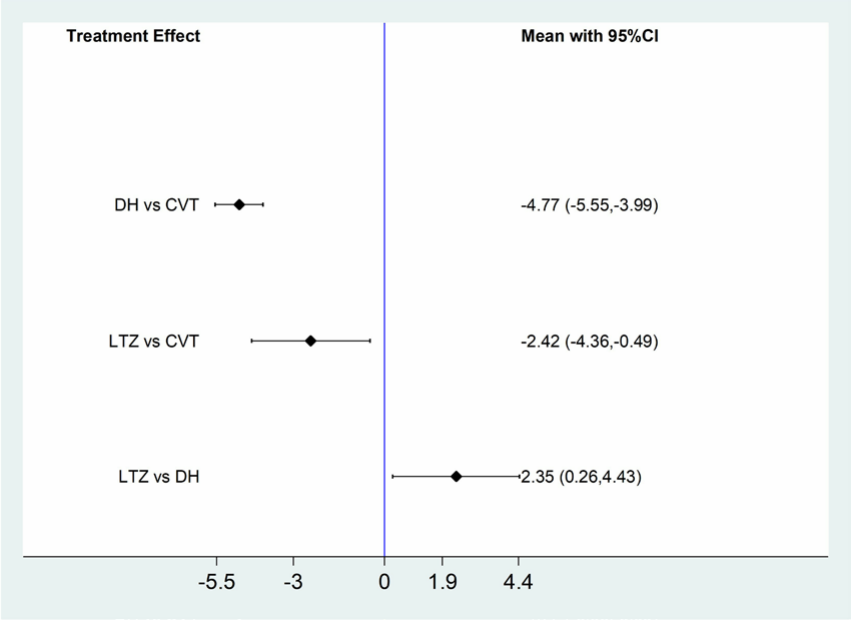
Comparative forest chart of PaCO_2_. PaCO2 = Partial pressure of carbon dioxide.

**Figure 15.**
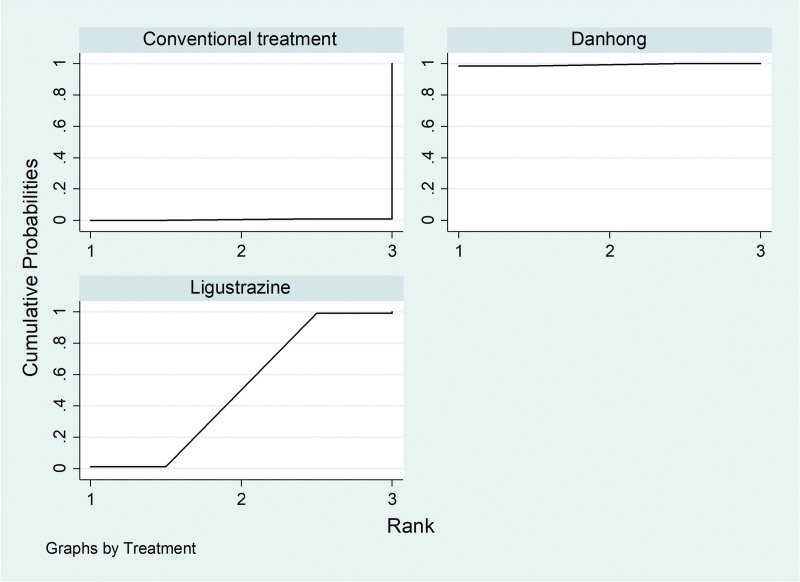
SUCRA ranking chart of PaCO_2_. PaCO_2_ = Partial pressure of carbon dioxide.

Among the 20 included literatures, 3 literatures reported the PaCO_2_ situation of IPF patients treated by Traditional Chinese medicine injection, among which Danhong injection treated IPF 1 article^[29]^ and ligustrazine injection treated IPF 2 articles.^[23,27]^According to the results of network meta-analysis with Stata14.0 software, the improvement of PaCO_2_ by Danhong injection (MD = −4.77, 95% CI [−5.55, −3.99], moderate certainty of evidence) and Ligustrazine injection (MD = −2.42, 95% CI [−4.36, −0.49], moderate certainty of evidence) was better than that of conventional treatment group, and the difference was statistically significant. Danhong injection (MD = −2.35, 95% CI [−4.43, −0.26], low certainty of evidence) had better efficacy than ligustrazine injection. From the SUCRA ranking order, Danhong (99.3) > Ligustrazine (50.3) > Conventional treatment (0.4). See Tables 12 and 13 and Figures 14 and 15 for details.

**Table 12:** NMA on PaCO_2_.

Danhong	2.35 (0.26, 4.43)	4.77 (3.99, 5.55)
	⊕⊕⊝⊝	⊕⊕⊕⊝
	Low^2^^,^^4^	Moderate^2^
−**2.35** (−**4.43,** −**0.26**)	Ligustrazine	2.42 (0.49, 4.36)
⊕⊕⊝⊝		⊕⊕⊕⊝
low^2^^,^^4^		Moderate^1^
−**4.77** (−**5.55,** −**3.99**)	−**2.42** (−**4.36,** −**0.49**)	Conventional
⊕⊕⊕⊝	⊕⊕⊕⊝	
Moderate^2^	Moderate^1^	

Note:

1 Certainty lowered for imprecision.

2 Certainty lowered for individual study risk of bias.

3 Certainty lowered two levels for imprecision.

4 Certainty lowered for indirectness.

GRADE Working Group grades of evidence – High quality: Further research is very unlikely to change our confidence in the estimate of effect; Moderate quality: Further research is likely to have an important impact on our confidence in the estimate of effect and may change the estimate; Low quality: Further research is very likely to have an important impact on our confidence in the estimate of effect and is likely to change the estimate; Very low quality: We are very uncertain about the estimate.

NMA = network meta analysis, PaCO2 = partial pressure of carbon dioxide.

**Table 13:** SUCRA value of PaCO_2_.

Treatment	SUCRA	PrBest	MeanRank
Conventional treatment	0.4	0.0	3.0
Danhong	99.3	98.6	1.0
Ligustrazine	50.3	1.4	2.0

NMA = network meta analysis, PaCO_2_ = partial pressure of carbon dioxide.

#### 3.4..4. Safety and adverse reactions

Among the 20 included studies, 14 did not describe adverse reactions, and 4^[21,22,30,31]^ indicated that there were no adverse reactions. The 4 include 2 studies of Danhong injection,^[21,30]^ 1 study of Huangqi injection,^[22]^ and 1 study of Dazhu Hongjingtian injection.^[31]^Two studies^[17,23]^ described adverse reactions and treatment methods, among which 1 study^[17]^ reported that 3 cases in the Danhong injection group and 3 cases in the conventional treatment group had conscious epigastric discomfort, which disappeared after adjusting the time of taking the Erythromycin Tablets to after meal (mainly considered as gastrointestinal stimulation caused by erythromycin), and no obvious adverse reactions were found in other groups. Another study^[23]^ described 7 cases of adverse reactions in the Ligustrazine group and the control group respectively, but the situation was relatively mild. The results are shown in Table 2. No significant adverse reactions were found in other cases.

#### 3.4..5. Publication bias

**Figure 16.**
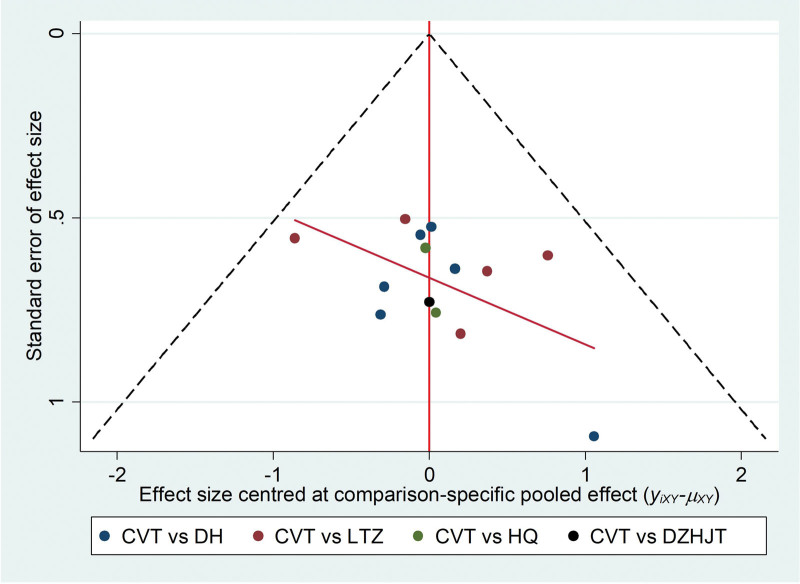
Inverted funnel chart of clinical efficacy.

**Figure 17.**
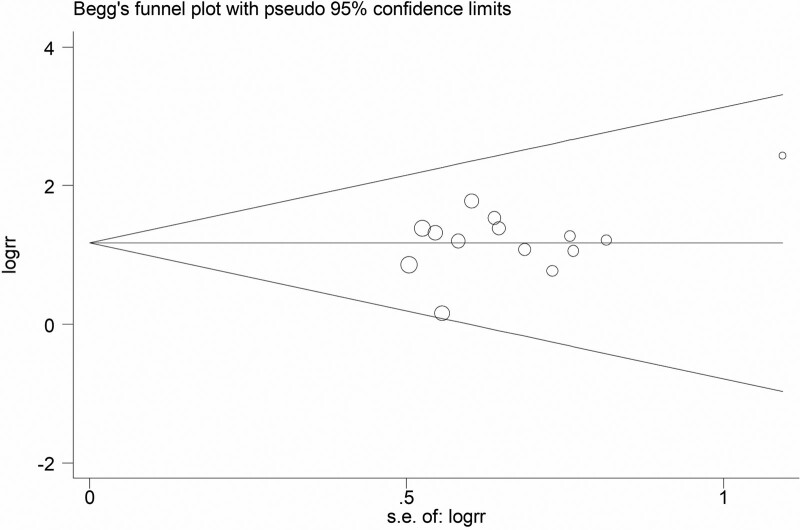
The funnel chart of bias generation detected by Begg rank correlation.

**Figure 18.**
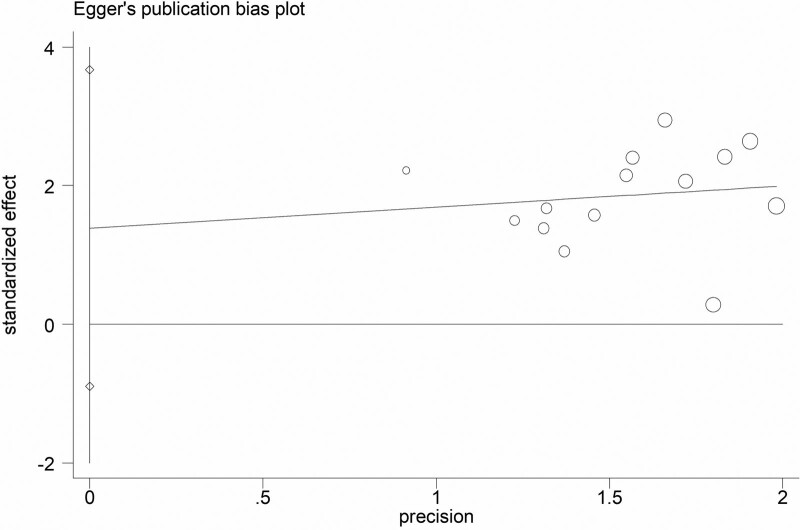
The funnel chart generated by Egger’s test.

The publication bias analysis was performed on the clinical efficacy data in the 14 studies. Figure 16 showed that the inverted funnel chart was symmetrical, which indicating that the results of this study are not biased but rather reliable. In addition, this systematic review also used the Begg’s Test and Egger’s test to detect the bias of the research results. The results of the Begg’s Test are shown in Table 14 and Figure 17 as *Pr*>|*z*|= 0.743, and the results of the Egger’s test are shown in Table 15 and Figure 18 as *P*>|*t*| = .211, these results have proved that the results of this study are not significantly biased.

**Table 14:** Detection results of bias in the study by Begg’s test.

Begg’s Test	
adj. Kendall’s Score (P–Q)	7
Std. Dev. of Score	18.27
Number of Studies	14
*z*	0.38
*Pr* > *z*	0.702
*z*	0.33 (continuity corrected)
*Pr* > *z*	0.743 (continuity corrected)

**Table 15:** Detection results of bias in the study by Egger’s test.

Egger’s test						
Std_Eff	Coef.	Std. Err.	*t*	*P*> *t*	[95% Confidence Interval]	
slope	0.3041649	0.6682947	0.46	.657	−1.151924	1.760254
bias	1.387358	1.049396	1.32	.211	−.8990794	3.673795

#### 3.4..6. Influence analysis

**Figure 19.**
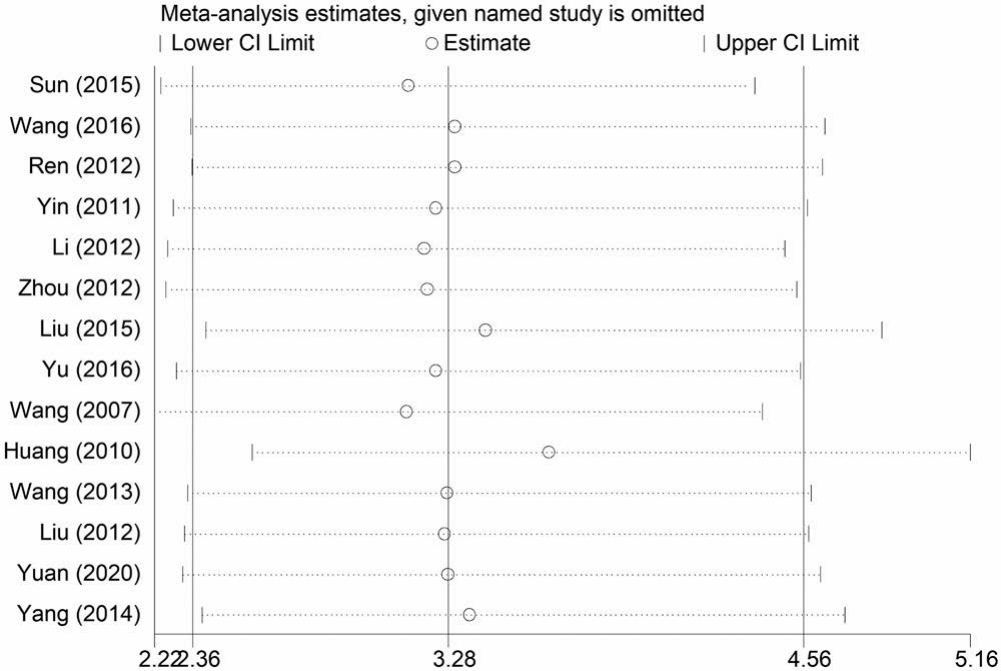
Influence analysis results of clinical efficacy.

The minimum value of all results of clinical efficacy of influence analysis was not lower than 1, indicating that the systematic review has good stability and reliability after excluding any one of the studies. This proves that the sensitivity of the results is low, and the analysis results are robust and reliable. The results are shown in Table 16 and Figure 19.

**Table 16:** Influence analysis results data of clinical efficacy.

Study omitted	Estimate	[95% Confidence Interval]	
Sun (2015)	3.1362484	2.2454355	4.380466
Wang (2016)	3.3014352	2.3522809	4.6335769
Ren (2012)	3.3014061	2.3570962	4.6240296
Yin (2011)	3.2351501	2.2902126	4.5699673
Li (2012)	3.1921325	2.2700427	4.4887748
Zhou (2012)	3.2033851	2.264092	4.5323586
Liu (2015)	3.411592	2.4063754	4.8367181
Yu (2016)	3.233839	2.3009439	4.5449672
Wang (2007)	3.1299226	2.2227099	4.4074202
Huang (2010)	3.6424172	2.5727007	5.1569161
Wang (2013)	3.2757609	2.3412583	4.5832663
Liu (2012)	3.2658162	2.3314154	4.5747123
Yuan (2020)	3.2761996	2.3243456	4.6178517
Yang (2014)	3.3559885	2.3934729	4.7055717
Combined	3.2796598	2.3609415	4.5558811

## 4. Discussion

This study included 20 RCTs on the clinical efficacy of 4 kinds of TCM injection combined with conventional treatment in the improvement of IPF. In general, 4 kinds of TCM injection combined with conventional treatment were effective in the improvement of IPF patients, which was shown to improve clinical effective rate, lung function (FEV_1_/FVC%, DLCO%) and the analysis of arterial blood gas (PaCO_2_, PaO_2_), and had certain safety. For the publication bias of the primary outcome indicators (clinical efficiency), the results of Begg’s test and Egger’s test showed that there was no bias, and the sensitivity analysis showed that the results were relatively robust and stable, which further proved that the results of this study were relatively reliable.

Danhong injection in this study contains the effective components of Salvia Miltiorrhiza (Danshen) and safflower (Honghua), both of which can promote blood circulation and remove blood stasis. The highest content of Salvia miltiorrhiza (Danshen) is Tanshinone IIA, which is one of the fat-soluble components extracted from Salvia miltiorrhiza (Danshen). 10 μmol/L Tanshinone IIA can reduce the expression of transforming growth factor-β1 (TGF-β_1_) receptor and the effect is significant.^[37]^ Experiments have shown that^[38]^ Tanshinone IIA can inhibit the signaling pathway of TGF-β_1_ and the key factor Smad2/3 in rats with pulmonary fibrosis. The main effective component of safflower (Honghua) is safflower yellow. Studies have found that^[39]^ safflower yellow can assist in the treatment of IPF and improve the life quality of patients, and delay the natural process of idiopathic pulmonary fibrosis. In addition, studies have found that^[40]^ Hydroxysafflor yellow A may play a role in alleviating cell damage caused by TGF-β_1_ through TGF-β_2_ receptors. The main component of Ligustrazine injection is Ligustrazine, which is an alkaloid monomer isolated and purified from (chuanxiong) Ligusticum wallichii. It is the main effective component of traditional Chinese medicine (chuanxiong)Ligusticum wallichii and can promote blood circulation to remove blood stasis. It has been reported^[41]^ that Ligustrazine has the effect of an antioxidant, effect of reducing oxidative damage, effect of reducing the secretion of cytokines, effect of reducing the expression of TGF-β_1_, and effect of inhibiting the division and proliferation of fibroblasts. In addition, meta analysis^[42]^ confirmed that Ligustrazine can treat IPF better. The main component of Huangqi injection is astragalus (Huangqi), which has the function of nourishing Qi in the lung. Astragalus contains active components such as Astragalus Polysaccharide, Astragalus Total Saponin and Astragalus Total Flavone etc. Modern research^[43]^ found that Astragalus Polysaccharides, Total Flavonoids and Total Saponins of Astragalus can interfere with alveolar inflammation in mice with pulmonary fibrosis caused by bleomycin, and have a certain therapeutic effect. The results of animal experiments showed that the number of inflammatory cells in BALF of Astragalus flavone treatment group was less than that of model group, which suggested that Astragalus flavonoids could antagonize inflammatory reaction of pulmonary fibrosis and inhibit the appearance of early fibrosis nodules. In addition, it has been reported in the literatures that astragaloside IV can inhibit the expression of CD34 and basic fibroblast growth factor in lung tissues of rats with pulmonary fibrosis, and the intervention effect is positively correlated with the dose.^[44,45]^ The main component of Dazhu Hongjingtian injection is Rhodiola macrophylla (Dazhu Hongjingtian), which can also promote blood circulation to remove blood stasis. Rhodiola macrophylla mainly contains salidroside and flavonoids, they have strong anti-fatigue, anti-hypoxia, anti-tumor, anti-aging, coronary artery expansion and other pharmacological effects.^[46]^ All the above results made a further proof that the 4 kinds of TCM injection are effective in the improvement of IPF.

### 4.1. Efficacy and adverse effects

The results of this network meta-analysis showed that Danhong (OR = 3.94, 95% CI [2.34, 6.64], moderate certainty of evidence), Huangqi (OR = 3.40, 95% CI [1.38, 8.41], moderate certainty of evidence) and Ligustrazine (OR = 2.74, 95% CI [1.62, 4.64], moderate certainty of evidence) combined with conventional treatment had obvious advantages in improving clinical efficiency; Danhong injection (MD = 12.25, 95% CI [9.60, 14.89], moderate certainty of evidence) combined with conventional treatment had the best effect in improving pulmonary function (FEV_1_/FVC%); For the improvement of DLCO%, Danhong injection (MD = 5.01, 95% CI [3.81, 6.21], moderate certainty of evidence) and Ligustrazine injection (MD = 9.12, 95% CI [5.70, 12.55], very low certainty of evidence) combined with conventional treatment both have good curative effect, of which Ligustrazine (MD = 4.11, 95% CI [0.49, 7.74], very low certainty of evidence) is the best; In terms of improving the analysis of blood gas (PaO_2_), Dazhu Hongjingtian injection (MD = 15.44, 95% CI [8.18, 22.70], moderate certainty of evidence), Danhong injection (MD = 14.15, 95% CI [12.58, 15.73], moderate certainty of evidence) and Ligustrazine injection (MD = 6.76, 95% CI [3.27, 10.26], moderate certainty of evidence) combined with conventional treatment showed obvious advantages compared with conventional treatment, among which Dazhu Hongjingtian injection and Danhong injection had the best therapeutic effect; In terms of improving PaCO_2_, compared with conventional treatment, the therapeutic effect of Danhong injection (MD = −4.77, 95% CI [−5.55, −3.99], moderate certainty of evidence) and Ligustrazine injection (MD = −2.42, 95% CI [−4.36, −0.49], moderate certainty of evidence)combined with conventional treatment is better, and Danhong injection (MD = −2.35, 95% CI [−4.43, −0.26], low certainty of evidence) is the best.

Among the 20 studies included, 2 cases reported adverse reactions, including 3 cases of epigastric discomfort in the treatment of Danhong injection, and 5 cases in Ligustrazine injection treating. However, the symptoms, time, and incidence of adverse reactions in patients are negligible, it was considered as the side effect of the combination of conventional treatment. Huangqi injection and Dazhu Hongjingtian injection showed no adverse reactions. But 14 studies did not mention the adverse reactions, and the safety and incidence of adverse reactions of four kinds of traditional Chinese medicine injection in the treatment of IPF have not been proved. In addition, the observation time of most studies wasn’t long (≤12 weeks), which makes it difficult to evaluate the long-term safety. Therefore, in terms of clinical efficacy and safety, Danhong injection has obvious advantages over other TCM injections in the treatment of IPF; Ligustrazine injection has a certain curative effect in improving clinical effective rate, lung function and the analysis of arterial blood gas, and it has obvious advantages in improving DLCO%, but the efficacy and safety of FEV_1_/FVC% need to be further determined; Dazhu Hongjingtian injection has a good effect in improving PaO_2_ of patients, but it has no obvious advantages in clinical efficiency and DLCO%, and there are few related reports, so its clinical efficacy and safety need to be further determined. Although Huangqi injection has a good effect in clinical efficiency, its treatment effect on secondary indicators of this study was less reported, so its effectiveness and safety need to be further clarified.

### 4.2. Research deficiencies

The 20 documents in this study were selected and included in strict accordance with the inclusion and exclusion criteria, but there were some shortcomings that need to be pointed out: 20 RCT studies all reported random methods, but three studies only mentioned random number table method, and did not give a clear information of how the random methods was generated, and the other 17 studies only mentioned random methods. No literature reports on allocation concealment and blinding, as well as the treatment of missing interviews. This result indicates that the literature included in this systematic review may be biased and the evidence strength is not high; Most studies only reported on the two indicators of clinical effectiveness and PaCO2, and the evaluation criteria of the indicators were not the same and the evaluation indicators were single. Therefore, there may be a certain degree of clinical heterogeneity in this systematic review; The researches were single centered with insufficient sample size. And there night be some certain clinical heterogeneities such as the course of treatment and different courses of disease. These reasons may decrease the reliability of this systematic review; Most of the literature in this study did not report the important clinical indicators such as Forced Expiratory Volume In 1s%, Forced vital capacity%, Total lung capacity, 6-min walking distance, St. George’s Respiratory Questionnaire, High-resolution computed tomography, inflammatory factors and pulmonary fibrosis factors, etc. These factors will lead to a single evaluation index in this systematic review and affect the credibility of clinical efficacy results; All the results reported in the literature in this study were conducted in China and not in other countries. These factors may lead to a large language bias, which may affect the reliability of the conclusion and extrapolation of the review; and No follow-up or long-term follow-up of cases was found in all studies, and the reported results were not evaluated, so the quality of the literature was medium.

## 5. Conclusion

In summary, TCM injections combined with conventional treatment can alleviate the clinical symptoms of IPF patients, increase lung function, improve arterial blood gas analysis, which shows that it is truly effective. This research result has 3 important meanings. First, it proves that the clinical efficacy of Danhong injection is better. It could be promoted and recommended in medical units around the world as a supplementary therapy. Second, based on the obvious clinical curative effect of Danhong injection, it can be further confirmed that blood stasis is the main cause of IPF, which exists in the whole process of the genesis and development of IPF. In the future, more clinical and animal studies can be invested on the pathological mechanism of IPF at this critical point. Third, compared with other TCM injections and conventional treatment, ligustrazine injection has certain advantages in the improvement of IPF, and it can be used according to the actual situation in clinic. Its clinical efficacy further proves that blood stasis is the main cause of IPF. However, the safety and adverse reactions of four kinds of TCM injections need to be further analyzed and determined. In addition, there are few clinical reports on Huangqi injection and Dazhu Hongjingtian injection, so the efficacy and safety of them cannot be proved yet. In the future, it’s necessary to have more high-quality RCTs to prove the effectiveness and safety of TCM injections, as well as its effect on mortality and other endpoint indicators. Further systematic reviews are needed to study more reliable conclusions to guide clinical practice considering the fact that there are not many relevant clinical research reports.

## Author contributions

This study is initiated by Xiaozheng Wu.

Xiaozheng Wu and Wen Li were involved in the design of the study and the interventions of the protocol.

Xiaozheng Wu will develop the search strategies, conduct data collection, and analyze independently.

Zhong Qin, Lei Xue, Zhenliang Luo and Yunzhi Chen will revise it.

All authors have approved the final manuscript.

**Conceptualization:** Xiaozheng Wu.

**Methodology:** Xiaozheng Wu, Wen Li.

**Software:** Xiaozheng Wu.

**Supervision:** Yunzhi Chen.

**Writing – original draft:** Xiaozheng Wu.

**Writing – review & editing:** Xiaozheng Wu, Wen Li, Yunzhi Chen.
